# 4-{3-[Hydr­oxy(phen­yl)meth­yl]-5-thioxo-4,5-dihydro-1*H*-1,2,4-triazol-4-yl}benzene­sulfonamide

**DOI:** 10.1107/S1600536810011402

**Published:** 2010-03-27

**Authors:** Mehmet Akkurt, Ísmail Çelik, Gökçe Cihan, Gültaze Çapan, Orhan Büyükgüngör

**Affiliations:** a Department of Physics, Faculty of Arts and Sciences, Erciyes University, 38039 Kayseri, Turkey; b Department of Physics, Faculty of Arts and Sciences, Cumhuriyet University, 58140 Sivas, Turkey; c Department of Pharmaceutical Chemistry, Faculty of Pharmacy, University of Istanbul, 34116 Beyazıt, Istanbul, Turkey; d Department of Physics, Faculty of Arts and Sciences, Ondokuz Mayıs University, 55139 Samsun, Turkey

## Abstract

In the title compound, C_15_H_14_N_4_O_3_S_2_, the hydr­oxy group is disordered over two positions with occupancies of 0.619 (5) and 0.381 (5). The benzene ring attached to the heterocycle makes a dihedral angle of 86.92 (9)° with respect to the best plane through the five-membered ring. The crystal packing is stabilized by inter­molecular O—H⋯O, N—H⋯S, N—H⋯N, C—H⋯O and C—H⋯N hydrogen bonds, and N—H⋯π and C—H⋯π inter­actions.

## Related literature

For the pharmacological activity of functionalized 1,2,4-triazoles, see: De La Rosa *et al.* (2006 ▶); Mavrova *et al.* (2009 ▶); Shiradkar *et al.* (2007 ▶). For annular tautomerism in 1,2,4-triazoles in the solid state and in solution, see: Buzykin *et al.* (2008 ▶); Dolzhenko *et al.* (2010 ▶). Two tautomeric fothione (C=S)r.m.s. for 3(5)-thioxo-1,2,4-triazoles may exist in the solid state. For the evidence for the thione (C=S) form, see: Karayel *et al.* (2007 ▶).
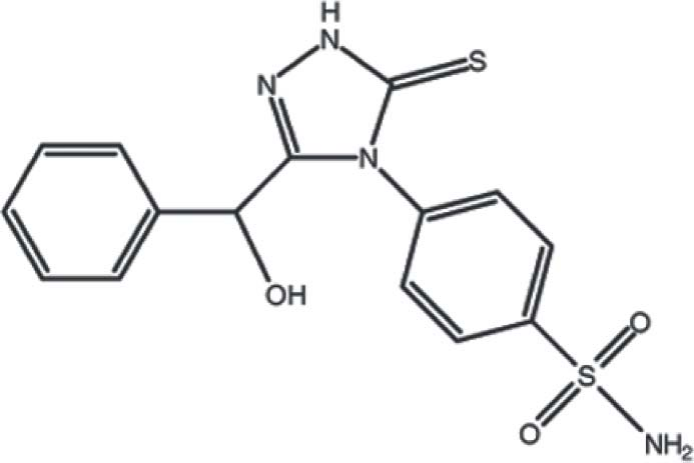


## Experimental

### 

#### Crystal data

C_15_H_14_N_4_O_3_S_2_
*M* *_r_* = 362.44
Orthorhombic, 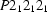

*a* = 8.2498 (5) Å
*b* = 13.5167 (7) Å
*c* = 14.2522 (7) Å
*V* = 1589.26 (15) Å^3^
*Z* = 4
Mo *K*α radiation
μ = 0.36 mm^−1^
*T* = 296 K
0.62 × 0.48 × 0.36 mm

#### Data collection

Stoe IPDS 2 diffractometer
Absorption correction: integration (*X-RED32*; Stoe & Cie, 2002 ▶) *T*_min_ = 0.815, *T*_max_ = 0.879
8693 measured reflections
3666 independent reflections
3050 reflections with *I* > 2σ(*I*)
*R*_int_ = 0.036

#### Refinement

*R*[*F* ^2^ > 2σ(*F* ^2^)] = 0.028
*wR*(*F* ^2^) = 0.066
*S* = 1.01
3666 reflections
245 parameters
4 restraints
H atoms treated by a mixture of independent and constrained refinement
Δρ_max_ = 0.22 e Å^−3^
Δρ_min_ = −0.22 e Å^−3^
Absolute structure: Flack (1983 ▶), 1538 Freidel pairs
Flack parameter: 0.14 (5)

### 

Data collection: *X-AREA* (Stoe & Cie, 2002 ▶); cell refinement: *X-AREA*; data reduction: *X-RED32* (Stoe & Cie, 2002 ▶); program(s) used to solve structure: *SIR97* (Altomare *et al.*, 1999 ▶); program(s) used to refine structure: *SHELXL97* (Sheldrick, 2008 ▶); molecular graphics: *ORTEP-3* (Farrugia, 1997 ▶); software used to prepare material for publication: *WinGX* (Farrugia, 1999 ▶).

## Supplementary Material

Crystal structure: contains datablocks global, I. DOI: 10.1107/S1600536810011402/bt5224sup1.cif

Structure factors: contains datablocks I. DOI: 10.1107/S1600536810011402/bt5224Isup2.hkl

Additional supplementary materials:  crystallographic information; 3D view; checkCIF report

## Figures and Tables

**Table 1:** Hydrogen-bond geometry (Å, °) *Cg*2 and *Cg*3 are centroids of the C1–C6 and C10–C15 rings, respectively.

*D*—H⋯*A*	*D*—H	H⋯*A*	*D*⋯*A*	*D*—H⋯*A*
O1*A*—H1*A*⋯O3^i^	0.82	2.35	2.973 (3)	134
N2—H2⋯S1^ii^	0.86	2.46	3.2744 (15)	158
N4—H4*A*⋯N1^iii^	0.88 (2)	2.18 (2)	3.052 (2)	171 (2)
C6—H6⋯O2^iv^	0.93	2.57	3.442 (2)	155
C12—H12⋯N1^v^	0.93	2.59	3.499 (2)	165
C15—H15⋯O2^iv^	0.93	2.48	3.2805 (19)	145
C2—H2*A*⋯*Cg*3^vi^	0.93	2.90	3.502 (2)	124
N4—H4*B*⋯*Cg*2^iii^	0.85 (2)	2.67 (2)	3.218 (2)	123 (2)
C14—H14⋯*Cg*2^vii^	0.93	2.83	3.463 (2)	126

Symmetry codes: (i) 
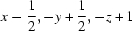
; (ii) 
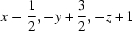
; (iii) 
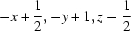
; (iv) 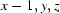; (v) 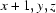; (vi) 
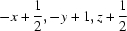
; (vii) 
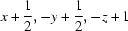
.

**Fig. 1.**
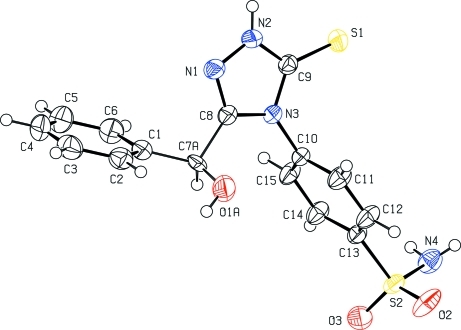
View of the title compound with the atom numbering scheme. Displacement ellipsoids for non-H atoms are drawn at the 50% probability level. The minor occupied sites of the disordered atoms have been omitted for clarity.

**Fig. 2.**
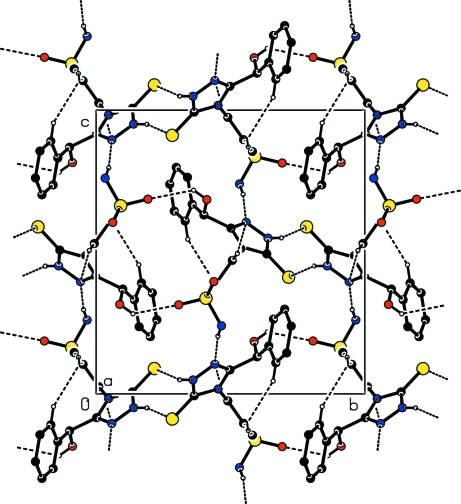
View down the a axis of the packing and hydrogen bonding interactions of the title compound. All hydrogen atoms not involved in hydrogen bonding and the minor occupied sites of the disordered atoms have been omitted for clarity.

## supplementary crystallographic information

### Comment

Functionalized 1,2,4-triazoles have attracted intense research interest after the discovery that  triazole have a wide range of pharmacological properties such as antiviral (De La Rosa *et al.*, 2006), anticancer (Mavrova *et al.*, 2009) and antimycobacterial activity (Shiradkar *et al.*, 2007).

Previous reports have also dealt with the annular tautomerism encountered in 1,2,4-triazoles in the solid state and in solution (Buzykin *et al.*, 2008; Dolzhenko *et al.*, 2010). For 3(5)-thioxo-1,2,4-triazoles, two tautomeric forms may exist in the solid state: the thione (C=S) and the thiol (SH), where the former has been supported by a recent X-ray diffraction study (Karayel *et al.*, 2007). In this context, we prepared the title compound to determine its pharmacological potential and the preferred tautomeric form in the solid state.

The title molecule (Fig. 1) the aromatic rings (C1–C6 and C10–C15) are oriented at angles of 73.79 (10) and 86.92 (9)° with respect to the best plane through the five-membered ring. The dihedral angle between the two six-membered rings is 47.58 (9)°.

In the structure, the molecules are linked by intermolecular O—H···O, N—H···S, N—H···N, C—H···O and C—H···N hydrogen bonds (Table 1, Fig. 2), and N—H···π and C—H···π interations (Table 1). In addition, there is also a weak S2—O3···Cg1 (-1+x, y, z) interaction [(S2)O3···Cg1 = 3.1082 (17) Å, S2—O3···Cg1 = 150.21 (9) °, where Cg1 is a centroid of the ring N1–N3/C8/C9].

### Experimental

A mixture of 1-[2-[hydroxy(phenyl)acetyl]]-4-(4-sulfamoylphenyl)-3-thiosemicarbazide (0.005 mol) and 2 N NaOH (20 ml) was heated on a water bath for 3 h. After cooling, the reaction mixture was acidified by the addition of HCl (% 12.5). The precipitate thus obtained was filtered, washed with water and recrystallized from aqueous ethanol.[Yield: 80.2 %, m.p.: 523-526 K]. IR (KBr) ν = 3518, 3437, 3346 (O—H, N—H), 1593 (C=N), 1342, 1154 (SO_2_) cm^-1^. ^1^H-NMR (DMSO-d_6_, 500 MHz) δ = 5.62 (1*H*, d, J=3.0 Hz, CHOH), 6.34 (1*H*, d, J=4.8 Hz, CHOH), 7.17-7.19 (2*H*, m, Ar—H), 7.22-7.25 (3*H*, m, Ar—H), 7.41 (2*H*, d, J=7.90 Hz, Ar—H), 7.52 (2*H*, s, SO_2_NH_2_), 7.87 (2*H*, d, J=8.54 Hz, Ar—H), 13.91 (1*H*, s, NH). Analysis calculated for C_15_H_14_N_4_O_3_S_2_ : C 49.71, H 3.89, N 15.46, S 17.69 %. Found : C 49.47, H 3.72, N 15.26, S 17.94 %.

### Refinement

The H atoms of the aromatic and hydroxyl groups were positioned geometrically with O—H = 0.82 Å, C—H = 0.93Å and refined using a riding model with *U*_iso_(H) = 1.2 or 1.5*U*_eq_(C,O). The H atoms of the NH_2_ group were located in a difference Fourier synthesis and their positional parameters were refined   with U_iso_(H) set to 1.2U_eq_(N). The N—H distances were restrained to 0.84 (1) Å. The H and O atoms of the hydroxyl group and the H atom of the C atom to which the hydroxyl group is attached are disordered in two alternative positions with occupancy factors 0.620 (4):0.380 (4). The C—O distances of the disordered hydroxyl group were restrained to 1.30 (1)Å.

### Crystal data

**Table:**

C_15_H_14_N_4_O_3_S_2_	*F*(000) = 752
*M**_r_* = 362.44	*D*_x_ = 1.515 Mg m^−^^3^
Orthorhombic, *P*2_1_2_1_2_1_	Mo *K*α radiation, λ = 0.71073 Å
Hall symbol: P 2ac 2ab	Cell parameters from 14591 reflections
*a* = 8.2498 (5) Å	θ = 1.4–28.0°
*b* = 13.5167 (7) Å	µ = 0.36 mm^−^^1^
*c* = 14.2522 (7) Å	*T* = 296 K
*V* = 1589.26 (15) Å^3^	Prism, colourless
*Z* = 4	0.62 × 0.48 × 0.36 mm

### Data collection

**Table:**

Stoe IPDS 2 diffractometer	3666 independent reflections
Radiation source: sealed X-ray tube, 12 x 0.4 mm long-fine focus	3050 reflections with *I* > 2σ(*I*)
plane graphite	*R*_int_ = 0.036
Detector resolution: 6.67 pixels mm^-1^	θ_max_ = 26.5°, θ_min_ = 2.1°
ω scans	*h* = −10→10
Absorption correction: integration (*X-RED32*; Stoe & Cie, 2002)	*k* = −14→16
*T*_min_ = 0.815, *T*_max_ = 0.879	*l* = −16→17
8693 measured reflections	

### Refinement

**Table:**

Refinement on *F*^2^	Secondary atom site location: difference Fourier map
Least-squares matrix: full	Hydrogen site location: inferred from neighbouring sites
*R*[*F*^2^ > 2σ(*F*^2^)] = 0.028	H atoms treated by a mixture of independent and constrained refinement
*wR*(*F*^2^) = 0.066	*w* = 1/[σ^2^(*F*_o_^2^) + (0.0413*P*)^2^] where *P* = (*F*_o_^2^ + 2*F*_c_^2^)/3
*S* = 1.01	(Δ/σ)_max_ = 0.001
3666 reflections	Δρ_max_ = 0.22 e Å^−^^3^
245 parameters	Δρ_min_ = −0.22 e Å^−^^3^
4 restraints	Absolute structure: Flack (1983), 1538 Freidel pairs
Primary atom site location: structure-invariant direct methods	Flack parameter: 0.14 (5)

### Special details

**Table:**

Geometry. Bond distances, angles etc. have been calculated using the rounded fractional coordinates. All su's are estimated from the variances of the (full) variance-covariance matrix. The cell esds are taken into account in the estimation of distances, angles and torsion angles
Refinement. Refinement on *F*^2^ for ALL reflections except those flagged by the user for potential systematic errors. Weighted *R*-factors wR and all goodnesses of fit *S* are based on *F*^2^, conventional *R*-factors *R* are based on *F*, with *F* set to zero for negative *F*^2^. The observed criterion of *F*^2^ > σ(*F*^2^) is used only for calculating -*R*-factor-obs etc. and is not relevant to the choice of reflections for refinement. *R*-factors based on *F*^2^ are statistically about twice as large as those based on *F*, and *R*-factors based on ALL data will be even larger.

### Fractional atomic coordinates and isotropic or equivalent isotropic displacement parameters (Å^2^)

**Table:**

	*x*	*y*	*z*	*U*_iso_*/*U*_eq_	Occ. (<1)
S1	0.17287 (5)	0.71648 (3)	0.41130 (3)	0.0371 (1)	
S2	0.79576 (4)	0.40875 (3)	0.33694 (3)	0.0315 (1)	
O1A	0.2506 (3)	0.4120 (2)	0.6848 (2)	0.0621 (9)	0.620 (4)
O2	0.92661 (14)	0.43807 (13)	0.39572 (10)	0.0510 (5)	
O3	0.77632 (18)	0.30741 (11)	0.31372 (14)	0.0592 (5)	
N1	−0.03407 (16)	0.55833 (11)	0.60444 (10)	0.0327 (4)	
N2	−0.01523 (16)	0.63968 (11)	0.54897 (10)	0.0317 (4)	
N3	0.18483 (14)	0.54616 (10)	0.51694 (9)	0.0270 (3)	
N4	0.81784 (19)	0.46661 (14)	0.23974 (11)	0.0406 (5)	
C1	−0.03337 (19)	0.35747 (13)	0.66854 (13)	0.0334 (5)	
C2	−0.0487 (2)	0.33998 (14)	0.76345 (13)	0.0382 (5)	
C3	−0.1902 (3)	0.29833 (14)	0.79838 (14)	0.0463 (6)	
C4	−0.3154 (2)	0.27399 (15)	0.73879 (17)	0.0496 (6)	
C5	−0.2995 (2)	0.29076 (16)	0.64419 (16)	0.0504 (6)	
C6	−0.1595 (2)	0.33125 (15)	0.60919 (14)	0.0426 (6)	
C7A	0.1213 (9)	0.4010 (5)	0.6305 (6)	0.0333 (16)	0.620 (4)
C8	0.08859 (17)	0.50199 (12)	0.58420 (12)	0.0295 (4)	
C9	0.11550 (16)	0.63506 (12)	0.49321 (12)	0.0282 (4)	
C10	0.33246 (16)	0.50982 (11)	0.47478 (11)	0.0264 (4)	
C11	0.47832 (19)	0.53833 (15)	0.51248 (13)	0.0369 (5)	
C12	0.62079 (19)	0.50687 (15)	0.47033 (14)	0.0379 (5)	
C13	0.61324 (17)	0.44786 (13)	0.39193 (12)	0.0289 (4)	
C14	0.46561 (18)	0.41826 (14)	0.35441 (12)	0.0353 (5)	
C15	0.32367 (18)	0.45127 (13)	0.39608 (13)	0.0352 (5)	
C7B	0.1184 (15)	0.4081 (11)	0.6206 (12)	0.055 (5)	0.380 (4)
O1B	0.2021 (5)	0.3451 (3)	0.5701 (3)	0.0600 (16)	0.380 (4)
H2A	0.03560	0.35610	0.80400	0.0460*	
H3	−0.20040	0.28680	0.86240	0.0560*	
H2	−0.08080	0.68910	0.54970	0.0380*	
H4A	0.734 (3)	0.4531 (19)	0.2033 (17)	0.0660*	
H4B	0.848 (3)	0.5258 (14)	0.251 (2)	0.0660*	
H4	−0.41020	0.24640	0.76250	0.0600*	
H6	−0.14900	0.34120	0.54490	0.0510*	
H7A	0.15510	0.35690	0.57950	0.0400*	0.620 (4)
H11	0.48150	0.57820	0.56560	0.0440*	
H12	0.72060	0.52560	0.49490	0.0450*	
H14	0.46200	0.37700	0.30220	0.0420*	
H15	0.22350	0.43400	0.37100	0.0420*	
H5	−0.38390	0.27460	0.60370	0.0600*	
H1A	0.26160	0.36280	0.71790	0.0930*	0.620 (4)
H1B	0.23780	0.30130	0.60420	0.0890*	0.380 (4)
H7B	0.19040	0.42150	0.67380	0.0660*	0.380 (4)

### Atomic displacement parameters (Å^2^)

**Table:**

	*U*^11^	*U*^22^	*U*^33^	*U*^12^	*U*^13^	*U*^23^
S1	0.0329 (2)	0.0357 (2)	0.0426 (2)	−0.0029 (2)	0.0009 (2)	0.0101 (2)
S2	0.0211 (2)	0.0390 (2)	0.0344 (2)	0.0051 (2)	0.0029 (1)	0.0016 (2)
O1A	0.0366 (11)	0.0597 (16)	0.090 (2)	−0.0082 (10)	−0.0184 (11)	0.0367 (15)
O2	0.0231 (5)	0.0929 (12)	0.0369 (7)	0.0062 (6)	−0.0020 (5)	0.0003 (7)
O3	0.0482 (7)	0.0366 (7)	0.0928 (12)	0.0067 (6)	0.0273 (8)	−0.0050 (8)
N1	0.0296 (6)	0.0344 (8)	0.0341 (7)	0.0042 (5)	0.0055 (5)	0.0036 (6)
N2	0.0286 (6)	0.0291 (7)	0.0375 (8)	0.0053 (5)	0.0045 (5)	0.0016 (6)
N3	0.0223 (5)	0.0278 (6)	0.0310 (7)	0.0012 (5)	0.0042 (5)	−0.0001 (5)
N4	0.0343 (7)	0.0527 (9)	0.0349 (8)	0.0026 (7)	0.0023 (6)	0.0022 (7)
C1	0.0311 (7)	0.0298 (8)	0.0392 (9)	0.0017 (6)	0.0023 (7)	0.0063 (8)
C2	0.0401 (9)	0.0372 (9)	0.0373 (9)	0.0029 (7)	−0.0039 (7)	0.0033 (8)
C3	0.0568 (11)	0.0421 (10)	0.0400 (10)	0.0060 (9)	0.0142 (9)	0.0081 (8)
C4	0.0392 (9)	0.0459 (11)	0.0637 (13)	−0.0063 (8)	0.0146 (9)	0.0049 (10)
C5	0.0405 (9)	0.0509 (11)	0.0598 (13)	−0.0063 (9)	−0.0057 (9)	−0.0014 (10)
C6	0.0446 (10)	0.0467 (11)	0.0366 (10)	−0.0007 (8)	−0.0020 (8)	0.0039 (8)
C7A	0.036 (3)	0.019 (2)	0.045 (3)	0.0050 (18)	0.006 (2)	0.015 (2)
C8	0.0253 (6)	0.0313 (8)	0.0318 (8)	0.0009 (5)	0.0035 (6)	0.0015 (7)
C9	0.0245 (6)	0.0292 (8)	0.0310 (8)	−0.0009 (6)	−0.0009 (6)	−0.0025 (7)
C10	0.0212 (6)	0.0271 (7)	0.0308 (7)	0.0014 (5)	0.0036 (5)	0.0016 (6)
C11	0.0278 (7)	0.0468 (10)	0.0362 (9)	0.0000 (7)	−0.0034 (6)	−0.0125 (8)
C12	0.0221 (7)	0.0498 (10)	0.0417 (10)	0.0002 (6)	−0.0043 (6)	−0.0110 (8)
C13	0.0221 (6)	0.0331 (8)	0.0315 (8)	0.0020 (5)	0.0029 (5)	0.0017 (7)
C14	0.0266 (7)	0.0436 (10)	0.0358 (9)	−0.0003 (7)	0.0021 (6)	−0.0123 (8)
C15	0.0215 (6)	0.0425 (9)	0.0417 (9)	−0.0028 (6)	0.0005 (6)	−0.0104 (8)
C7B	0.019 (5)	0.084 (11)	0.063 (8)	0.008 (5)	0.008 (4)	0.007 (7)
O1B	0.069 (3)	0.044 (2)	0.067 (3)	0.0257 (19)	0.044 (2)	0.0191 (19)

### Geometric parameters (Å, °)

**Table:**

S1—C9	1.6727 (17)	C3—C4	1.377 (3)
S2—O2	1.4227 (14)	C4—C5	1.374 (3)
S2—O3	1.4183 (16)	C5—C6	1.372 (3)
S2—N4	1.6012 (17)	C7A—C8	1.540 (7)
S2—C13	1.7779 (15)	C7B—C8	1.393 (15)
O1A—C7A	1.326 (8)	C10—C11	1.373 (2)
O1B—C7B	1.312 (16)	C10—C15	1.375 (2)
O1A—H1A	0.8200	C11—C12	1.387 (2)
O1B—H1B	0.8200	C12—C13	1.374 (3)
N1—C8	1.299 (2)	C13—C14	1.389 (2)
N1—N2	1.363 (2)	C14—C15	1.387 (2)
N2—C9	1.341 (2)	C2—H2A	0.9300
N3—C8	1.381 (2)	C3—H3	0.9300
N3—C9	1.373 (2)	C4—H4	0.9300
N3—C10	1.4442 (18)	C5—H5	0.9300
N2—H2	0.8600	C6—H6	0.9300
N4—H4A	0.88 (2)	C7A—H7A	0.9800
N4—H4B	0.85 (2)	C7B—H7B	0.9800
C1—C2	1.379 (3)	C11—H11	0.9300
C1—C7A	1.506 (8)	C12—H12	0.9300
C1—C6	1.387 (2)	C14—H14	0.9300
C1—C7B	1.582 (14)	C15—H15	0.9300
C2—C3	1.388 (3)		
			
S1···N2^i^	3.2744 (15)	C8···O2^xii^	3.122 (2)
S1···O3^ii^	3.460 (2)	C9···O2^xii^	3.384 (2)
S1···H2A^iii^	3.0100	C10···O1A	3.341 (3)
S1···H1A^iii^	3.0100	C10···O1B	2.821 (4)
S1···H2^i^	2.4600	C12···C6^v^	3.583 (3)
S1···H4^iv^	3.0700	C13···C2^iii^	3.444 (3)
S2···H6^v^	3.1300	C14···C6^vi^	3.565 (3)
S2···H1B^vi^	3.0000	C14···C5^vi^	3.573 (3)
O1A···N3	3.051 (3)	C14···C2^iii^	3.582 (3)
O1A···C10	3.341 (3)	C15···O1B	3.036 (5)
O1A···O3^vii^	2.973 (3)	C15···C5^vi^	3.473 (3)
O1B···O3^vii^	2.714 (4)	C15···O2^xii^	3.2805 (19)
O1B···C15	3.036 (5)	C1···H4A^x^	3.09 (3)
O1B···N3	2.825 (4)	C2···H1A	2.6600
O1B···C10	2.821 (4)	C2···H4B^x^	3.07 (2)
O2···N3^v^	3.1076 (19)	C2···H14^vii^	3.0800
O2···C15^v^	3.2805 (19)	C3···H14^vii^	3.0400
O2···C8^v^	3.122 (2)	C3···H4B^x^	2.79 (2)
O2···C9^v^	3.384 (2)	C4···H4B^x^	2.725 (19)
O3···C7A^vi^	3.194 (7)	C5···H15^vii^	3.0500
O3···C1^vi^	3.400 (2)	C5···H4B^x^	2.94 (2)
O3···C7B^vi^	3.326 (15)	C8···H6	2.9800
O3···O1B^vi^	2.714 (4)	C9···H3^xiii^	2.9900
O3···S1^viii^	3.460 (2)	C10···H7A	2.9400
O3···O1A^vi^	2.973 (3)	H1A···S1^x^	3.0100
O1A···H2A	2.5700	H1A···C2	2.6600
O1B···H6	2.9200	H1A···H2A	2.2300
O2···H12	2.5100	H1A···O3^vii^	2.3500
O2···H15^v^	2.4800	H1B···O3^vii^	1.9000
O2···H6^v^	2.5700	H1B···S2^vii^	3.0000
O3···H7A^vi^	2.8700	H2···S1^xi^	2.4600
O3···H14	2.7600	H2A···O1A	2.5700
O3···H1B^vi^	1.9000	H2A···S1^x^	3.0100
O3···H1A^vi^	2.3500	H2A···H1A	2.2300
O3···H4^ix^	2.9000	H2A···H7B	2.4200
N1···N3	2.2008 (18)	H3···C9^xiv^	2.9900
N1···C6	3.240 (2)	H4···O3^xv^	2.9000
N1···N4^x^	3.052 (2)	H4···S1^xvi^	3.0700
N2···N3	2.1285 (19)	H4A···C1^iii^	3.09 (3)
N2···S1^xi^	3.2744 (15)	H4A···N1^iii^	2.18 (2)
N3···O1B	2.825 (4)	H4B···C2^iii^	3.07 (2)
N3···N2	2.1285 (19)	H4B···C3^iii^	2.79 (2)
N3···O1A	3.051 (3)	H4B···C5^iii^	2.94 (2)
N3···O2^xii^	3.1076 (19)	H4B···C4^iii^	2.725 (19)
N4···C2^iii^	3.447 (3)	H6···S2^xii^	3.1300
N4···N1^iii^	3.052 (2)	H6···O2^xii^	2.5700
N4···C3^iii^	3.450 (3)	H6···C8	2.9800
N1···H12^xii^	2.5900	H6···H7A	2.5700
N1···H4A^x^	2.18 (2)	H6···O1B	2.9200
N2···H12^xii^	2.7800	H7A···C10	2.9400
C1···O3^vii^	3.400 (2)	H7A···H6	2.5700
C2···C13^x^	3.444 (3)	H7A···O3^vii^	2.8700
C2···N4^x^	3.447 (3)	H7B···H2A	2.4200
C2···C14^x^	3.582 (3)	H12···N2^v^	2.7800
C3···N4^x^	3.450 (3)	H12···N1^v^	2.5900
C5···C15^vii^	3.473 (3)	H12···O2	2.5100
C5···C14^vii^	3.573 (3)	H14···C2^vi^	3.0800
C6···C12^xii^	3.583 (3)	H14···O3	2.7600
C6···C14^vii^	3.565 (3)	H14···C3^vi^	3.0400
C6···N1	3.240 (2)	H15···C5^vi^	3.0500
C7A···O3^vii^	3.194 (7)	H15···O2^xii^	2.4800
C7B···O3^vii^	3.326 (15)		
			
O2—S2—O3	119.49 (10)	N3—C8—C7A	125.5 (3)
O2—S2—N4	106.68 (9)	S1—C9—N2	127.62 (12)
O2—S2—C13	107.47 (8)	S1—C9—N3	129.04 (11)
O3—S2—N4	106.42 (11)	N2—C9—N3	103.28 (14)
O3—S2—C13	107.11 (9)	N3—C10—C11	118.74 (14)
N4—S2—C13	109.42 (8)	C11—C10—C15	121.81 (14)
C7A—O1A—H1A	109.00	N3—C10—C15	119.39 (12)
C7B—O1B—H1B	109.00	C10—C11—C12	119.16 (17)
N2—N1—C8	104.79 (13)	C11—C12—C13	119.46 (15)
N1—N2—C9	113.44 (14)	S2—C13—C12	119.51 (11)
C9—N3—C10	123.12 (13)	S2—C13—C14	119.16 (13)
C8—N3—C9	108.06 (12)	C12—C13—C14	121.33 (14)
C8—N3—C10	128.82 (13)	C13—C14—C15	118.87 (16)
N1—N2—H2	123.00	C10—C15—C14	119.35 (14)
C9—N2—H2	123.00	C3—C2—H2A	120.00
H4A—N4—H4B	122 (2)	C1—C2—H2A	120.00
S2—N4—H4A	108.6 (16)	C2—C3—H3	120.00
S2—N4—H4B	109.2 (19)	C4—C3—H3	120.00
C2—C1—C7B	124.8 (6)	C3—C4—H4	120.00
C2—C1—C7A	119.9 (3)	C5—C4—H4	120.00
C6—C1—C7A	121.1 (4)	C6—C5—H5	120.00
C6—C1—C7B	116.2 (6)	C4—C5—H5	120.00
C2—C1—C6	119.05 (16)	C5—C6—H6	120.00
C1—C2—C3	119.89 (17)	C1—C6—H6	120.00
C2—C3—C4	120.42 (19)	O1A—C7A—H7A	106.00
C3—C4—C5	119.63 (17)	C8—C7A—H7A	106.00
C4—C5—C6	120.21 (18)	C1—C7A—H7A	106.00
C1—C6—C5	120.78 (19)	C8—C7B—H7B	103.00
O1A—C7A—C8	107.0 (5)	C1—C7B—H7B	103.00
O1A—C7A—C1	121.0 (6)	O1B—C7B—H7B	103.00
C1—C7A—C8	110.6 (5)	C10—C11—H11	120.00
O1B—C7B—C8	118.7 (12)	C12—C11—H11	120.00
C1—C7B—C8	114.5 (8)	C11—C12—H12	120.00
O1B—C7B—C1	111.9 (10)	C13—C12—H12	120.00
N1—C8—N3	110.41 (14)	C15—C14—H14	121.00
N1—C8—C7A	124.1 (3)	C13—C14—H14	121.00
N3—C8—C7B	123.4 (6)	C10—C15—H15	120.00
N1—C8—C7B	126.1 (6)	C14—C15—H15	120.00
			
O2—S2—C13—C12	7.66 (18)	C2—C1—C6—C5	1.6 (3)
O2—S2—C13—C14	−172.54 (15)	C7A—C1—C6—C5	179.4 (3)
O3—S2—C13—C12	137.22 (16)	C2—C1—C7A—O1A	8.1 (7)
O3—S2—C13—C14	−42.98 (18)	C2—C1—C7A—C8	−118.0 (4)
N4—S2—C13—C12	−107.81 (16)	C6—C1—C7A—O1A	−169.6 (4)
N4—S2—C13—C14	71.99 (16)	C6—C1—C7A—C8	64.3 (6)
C8—N1—N2—C9	0.93 (18)	C1—C2—C3—C4	0.2 (3)
N2—N1—C8—N3	0.02 (18)	C2—C3—C4—C5	0.3 (3)
N2—N1—C8—C7A	179.2 (4)	C3—C4—C5—C6	0.2 (3)
N1—N2—C9—S1	175.96 (12)	C4—C5—C6—C1	−1.2 (3)
N1—N2—C9—N3	−1.44 (18)	O1A—C7A—C8—N1	−109.8 (5)
C9—N3—C8—N1	−0.90 (18)	O1A—C7A—C8—N3	69.3 (6)
C9—N3—C8—C7A	179.9 (4)	C1—C7A—C8—N1	23.9 (7)
C10—N3—C8—N1	179.74 (14)	C1—C7A—C8—N3	−157.0 (3)
C10—N3—C8—C7A	0.6 (4)	N3—C10—C11—C12	−177.57 (16)
C8—N3—C9—S1	−175.99 (13)	C15—C10—C11—C12	−0.3 (3)
C8—N3—C9—N2	1.37 (17)	N3—C10—C15—C14	178.55 (15)
C10—N3—C9—S1	3.4 (2)	C11—C10—C15—C14	1.3 (3)
C10—N3—C9—N2	−179.23 (13)	C10—C11—C12—C13	−0.1 (3)
C8—N3—C10—C11	−94.4 (2)	C11—C12—C13—S2	179.21 (15)
C8—N3—C10—C15	88.3 (2)	C11—C12—C13—C14	−0.6 (3)
C9—N3—C10—C11	86.3 (2)	S2—C13—C14—C15	−178.22 (14)
C9—N3—C10—C15	−90.99 (19)	C12—C13—C14—C15	1.6 (3)
C6—C1—C2—C3	−1.2 (3)	C13—C14—C15—C10	−1.9 (3)
C7A—C1—C2—C3	−178.9 (3)		

Symmetry codes: (i) *x*+1/2, −*y*+3/2, −*z*+1; (ii) −*x*+1, *y*+1/2, −*z*+1/2; (iii) −*x*+1/2, −*y*+1, *z*−1/2; (iv) −*x*−1/2, −*y*+1, *z*−1/2; (v) *x*+1, *y*, *z*; (vi) *x*+1/2, −*y*+1/2, −*z*+1; (vii) *x*−1/2, −*y*+1/2, −*z*+1; (viii) −*x*+1, *y*−1/2, −*z*+1/2; (ix) *x*+3/2, −*y*+1/2, −*z*+1; (x) −*x*+1/2, −*y*+1, *z*+1/2; (xi) *x*−1/2, −*y*+3/2, −*z*+1; (xii) *x*−1, *y*, *z*; (xiii) −*x*, *y*+1/2, −*z*+3/2; (xiv) −*x*, *y*−1/2, −*z*+3/2; (xv) *x*−3/2, −*y*+1/2, −*z*+1; (xvi) −*x*−1/2, −*y*+1, *z*+1/2.

### Hydrogen-bond geometry (Å, °)

**Table:**

Cg2 and Cg3 are centroids of the C1–C6 and C10–C15 rings, respectively.

**Table:**

*D*—H···*A*	*D*—H	H···*A*	*D*···*A*	*D*—H···*A*
O1A—H1A···O3^vii^	0.82	2.35	2.973 (3)	134
N2—H2···S1^xi^	0.86	2.46	3.2744 (15)	158
N4—H4A···N1^iii^	0.88 (2)	2.18 (2)	3.052 (2)	171 (2)
C2—H2A···O1A	0.93	2.57	2.881 (3)	100
C6—H6···O2^xii^	0.93	2.57	3.442 (2)	155
C12—H12···O2	0.93	2.51	2.892 (2)	105
C12—H12···N1^v^	0.93	2.59	3.499 (2)	165
C15—H15···O2^xii^	0.93	2.48	3.2805 (19)	145
C2—H2A···Cg3^x^	0.93	2.90	3.502 (2)	124
N4—H4B···Cg2^iii^	0.85 (2)	2.67 (2)	3.218 (2)	123 (2)
C14—H14···Cg2^vi^	0.93	2.83	3.463 (2)	126

Symmetry codes: (vii) *x*−1/2, −*y*+1/2, −*z*+1; (xi) *x*−1/2, −*y*+3/2, −*z*+1; (iii) −*x*+1/2, −*y*+1, *z*−1/2; (xii) *x*−1, *y*, *z*; (v) *x*+1, *y*, *z*; (x) −*x*+1/2, −*y*+1, *z*+1/2; (vi) *x*+1/2, −*y*+1/2, −*z*+1.
